# Metronomic chemotherapy using capecitabine and cyclophosphamide in metastatic breast cancer – efficacy, tolerability and quality of life results from the phase II METRO trial

**DOI:** 10.1016/j.breast.2024.103795

**Published:** 2024-09-01

**Authors:** Karolina Larsson, Jamila Adra, Leif Klint, Barbro Linderholm

**Affiliations:** a Department of Oncology, Sahlgrenska University Hospital, Sweden; b Institute of Clinical Sciences, Sahlgrenska Academy, University of Gothenburg, Sweden

**Keywords:** Metastatic breast cancer, Metronomic chemotherapy, Quality of life

## Abstract

**Background:**

Chemotherapy is commonly used in metastatic breast cancer (MBC) to prolong life and improve quality of life (QoL). The optimal dosing and sequencing beyond the second line of treatment are unknown and pose a risk of overtreatment. Continuous low oral doses of metronomic chemotherapy using capecitabine 500 mg three times daily and cyclophosphamide 50 mg once daily (MCT-CX) may be an effective and tolerable treatment option for patients with MBC.

**Methods:**

In this open-label, single-arm single-centre phase II trial patients with MBC received MCT-CX until disease progression or unacceptable toxicity. The primary endpoint was the clinical benefit rate (CBR), defined as the proportion of participants with a best overall response of complete (CR) or partial response (PR) at any time, or stable disease (SD) for ≥24 weeks according to radiological evaluation. Toxicity was assessed according to the Common Toxicity Criteria v 4.0. QoL was assessed with the EORTC-30 questionnaire.

**Results:**

In total, 40 patients were included. Most participants (72 %) presented with visceral disease and received MCT-CX beyond the second line (58 %). The CBR was 45 % (8 PR and 10 SD ≥ 24 weeks). Toxicities were low grade with hand-foot syndrome being the most common. There was no significant change in QoL over the first 24 weeks.

**Conclusion:**

MCT-CX is a plausible treatment option in far advanced breast cancer, with almost half of trial participants responding to treatment without QoL impairments.

## Highlights

• Patients with metastatic breast cancer prefer convinient treatments but without impaired efficiacy.
• For most patients, chemotherapy emerges as a treatment option along the treatment pathway.
• Metronomic chemotherapy with capecitabine and cyclophosphamide is a plausible treatment option with low toxicity.

## Graphical abstract

**Figure:**
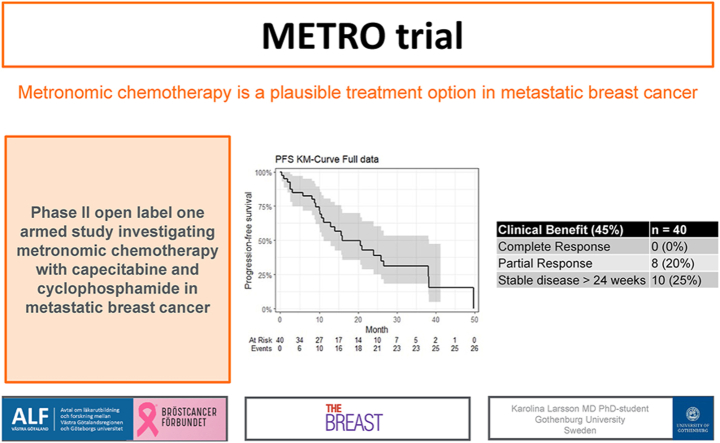

## 1 Introduction

Metastatic breast cancer (MBC) is with few exceptions an incurable disease with a median overall survival (OS) of around three years [1]. Treatment recommendations are based on breast cancer subtype, patient-related factors, and the preferences of the individual patient [2]. Where there is access to care, most patients with MBC are usually under oncological treatment continuously or intermittently until death. Systemic oncological treatment options for MBC include endocrine therapy, chemotherapy, and targeted therapies. In luminal breast cancer endocrine treatments are usually used in first and second line. The introduction of CDK4/6 inhibitors has improved outcomes delaying endocrine resistance [3,4] meaning that the time until treatment with chemotherapy is needed can be delayed. However, for most patients with MBC, chemotherapy emerges as an option along the treatment pathway. Since there is limited evidence comparing treatment strategies in MBC, especially beyond the second line, there is a problem of over-treatment that can impair wellbeing. This could be due to a view – held by the treating oncologist and/or the patient that more aggressive treatment is preferable [5]. It has been shown that combination chemotherapies can increase progression survival but with increased toxicity and no survival benefit compared to sequential treatment [6]. This indicates that more treatment isn't always better.

Metronomic chemotherapy (MCT) is the administration of continuously low doses of oral chemotherapy in contrast to the administration of chemotherapy at the maximum tolerated dose (MTD) [7]. MCT is recommended in patients not requiring a rapid tumour response [2], typically in frail patients, and can apply to different chemotherapeutic agents. The recently published (2023) randomized controlled trial (RCT), METEORA, showed favourable outcomes for MCT compared to weekly paclitaxel in endocrine-resistant luminal MBC [8]. However, since the metronomic regimen in METEORA contained three chemotherapeutic agents and there was a higher frequency of adverse events with MCT, one might argue that it was not strictly a low-dose regimen [8]. While the promising results of METEORA suggest that MCT could be a beneficial treatment strategy in MBC, the optimal treatment schedule has not yet been determined.

The purpose of this study was to evaluate the efficacy and tolerability of metronomic treatment with capecitabine and cyclophosphamide (MCT-CX) in MBC as well as the impacts on QoL.

## 2 Materials and methods

### 2.1 Study design

The METRO-trial was a prospective, open-label, single arm phase II study with 40 participants conducted at a single site in Sweden, between March 2019 and October 2023. ClinicalTrials.gov ID NCT04350021.

### 2.2 Patients and treatment

Patients were eligible for inclusion if they had incurable locally advanced or metastatic breast cancer, all subtypes and treatment lines were allowed. The full list of inclusion and exclusion criteria can be found in the Supplementary Methods.

Study participants were treated continuously with MCT-CX: oral capecitabine (500 mg three times daily) and cyclophosphamide (50 mg once daily). Participants with HER2-positive breast cancer received concomitant treatment with HER2-agents. Dose reductions were performed according to local routine. Treatment continued until disease progression, unaccepted toxicity, or death. Radiotherapy was administered for local symptom control if needed and supportive care was given concomitant to MCT-CX.

### 2.3 Outcomes

The participants were evaluated every twelve weeks or earlier if clinically indicated. The primary endpoint was the clinical benefit rate (CBR), defined as the proportion of participants with radiological complete (CR) or partial response (PR) at any time or stable disease (SD) for ≥24 weeks determined by the Response Evaluation Criteria in Solid Tumours (RECIST) guidelines [9]. Participants with skeleton-only disease could reach SD as the best effect. Secondary endpoints were over all response rate (ORR), defined as CR or PR at any time, progression-free survival (PFS), overall survival (OS), toxicity using the Common Toxicity Criteria (CTC) v 4.0, and patient-reported QoL measured with the European Organisation for Research and Treatment of Cancer Quality of Life-Core30 (EORTC – QLQ-30) questionnaire. Quality of life was measured at baseline and repeated every twelve weeks until progression.

### 2.4 Statistical analysis

A Fleming two-stage design was applied [10]. A total of 40 patients were planned for inclusion to obtain a minimum of 35 evaluable cases. The analysis of efficacy was based on the proportion of responders (i.e.,CBR). The null hypothesis (H0) was a CBR of 20 % (clinically irrelevant) and the alternative hypothesis (H1) was a CBR of 35 % (clinically successful). Based on two rounds of testing, the complete sample of 40 participants would be needed to reach a significance level (α) of 0.05 and a power of 80 %. The study could end at stage one after 20 evaluable participants if there was a clinical benefit in 8 participants (treatment considered futile) or ≥ 13 participants (treatment considered successful). At the completion of the second stage treatment would be considered successful if there were more than 10 responders. Progression-free-survival (PFS) and Overall-survival (OS) were evaluated using the Kaplan-Meier method. Baseline characteristics and toxicities were presented descriptively. The Wilcoxon test was used to compare outcomes of QoL assessments between the different time points (baseline, after 12 weeks, and after 24 weeks) with a significance level of 0.05. After discontinuation of treatment, participants were followed for survival until the data cut-off date on 31 October 2023. All statistical analyses were performed using the statistical software R version 4.2.2.

### 2.5 Ethics

The study protocol was approved by the regional ethical review board in Gothenburg (Dnr 875-18). Signed and dated informed consent was obtained from each participant in accordance with the Declaration of Helsinki and with the principles of Good Clinical Practice.

## 3 Results

### 3.1 Participants

The METRO-trial enrolled 40 patients (39 female and one male) between 3 March 2019, and 28 March 2023. The median age was 68 years (range 30–80), and 36 participants (92 %) were postmenopausal. All subtypes were represented with 33 participants (82 %) having luminal breast cancer. Visceral metastases were present in 29 participants (72 %). Twenty-three (58 %) participants received treatment beyond the second line and 26 participants had prior treatment with CDK4/6 inhibitors in the luminal group. Demographics are shown in Table 1. Among the 40 included participants, one withdrew consent and three were excluded due to protocol violations. At the cutoff date on 31 October 2023 all but two participants had discontinued treatment, and 24 (60 %) had died.

**Table 1:** Patient and tumour characteristics in a cohort of 40 patients with metastatic breast cancer treated with metronomic chemotherapy with capecitabine and cyclophosphamide.

Characteristics	Participants n = 40
Sex	
Female	39/40 (98 %)
Male	1/40 (2.5 %)
BMI	24.4 (4.3); 23.7 (7.0); (17.5–32.9)
Menopausal status	
Post	36/39 (92 %)
Pre	3/39 (7.7 %)
NA	1
ECOG performance status	
0	13/39 (33 %)
1	14/39 (36 %)
2	12/39 (31 %)
NA	1
Tumour subtype	
HER2-positive	2/40 (5.0 %)
Luminal	33/40 (82 %)
TNBC	5/40 (12 %)
Treatment Line	
1st line	4/40 (10 %)
2nd line	13/40 (32 %)
3rd line	14/40 (35 %)
4th line	7/40 (18 %)
5th line	2/40 (5.0 %)
Metastasic sites	
Bone only disease	7/40 (18 %)
Skin related disease	4/40 (10 %)
Visceral disease	29/40 (72 %)

^1^n/N (%); Mean (SD); Median (IQR); (Minimum-Maximum).

### 3.2 Clinical outcomes

The first analysis was conducted based on 20 evaluable participants. Ten had a CB, four with PR, and six with SD for ≥24 weeks, predefined criteria were met to continue the trial. The second analysis based on all 40 included patients showed a CB in 18 participants: eight with PR and ten with SD for ≥24 weeks giving a CBR of 45 %. (Table 2). The ORR was 20 %, with eight PR. Notably, 15 (38 %) participants had non-measurable disease and therefore could only reach SD as a best outcome.

**Table 2:** Clinical benefit rate in a cohort of 40 patients with metastatic breast cancer treated with metronomic chemotherapy with capecitabine and cyclophosphamide.

Outcome	N	Percent
CBR	18	45 %
NO CBR	22	55 %

**Fig. 1:**
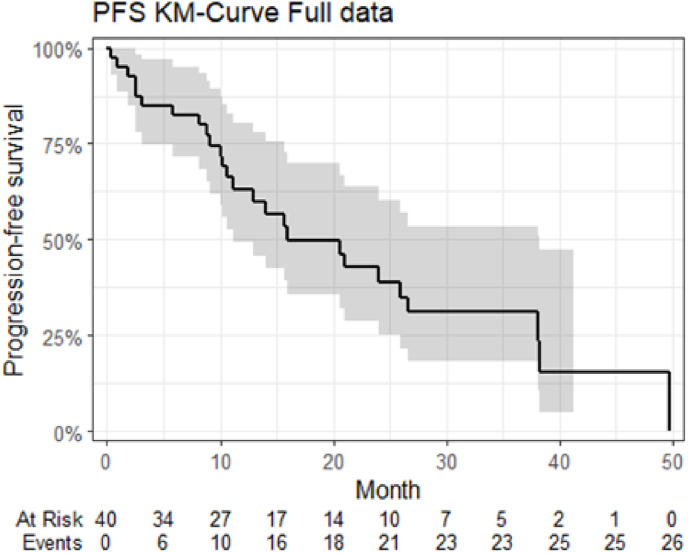
PFS in a cohort of 40 patients with metastatic breast cancer treated with metronomic chemotherapy with capecitabine and cyclophosphamide.

**Fig. 2:**
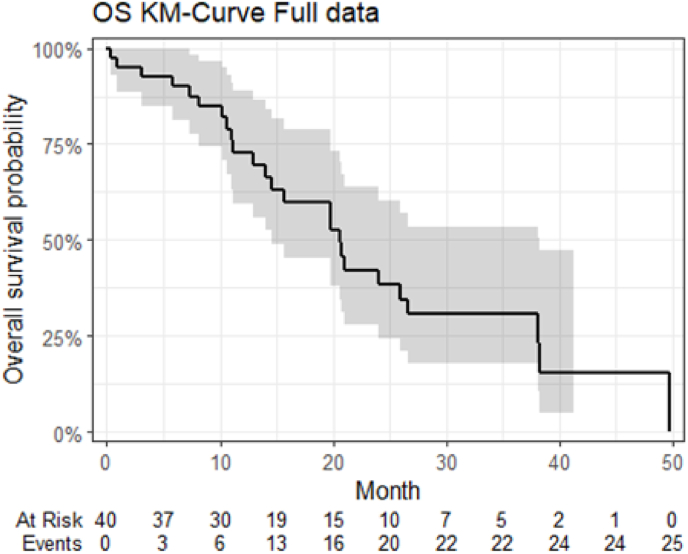
OS in a cohort of 40 patients with metastatic breast cancer treated with metronomic chemotherapy with capecitabine and cychlophosphamide

None of the participants with triple negative and HER2-positive breast cancer responded to treatment. Among those with the luminal subtype, where 25 (76 %) were classified as luminal B-like, 18 (55 %) responded to treatment. Out of 20 participants with liver metastasis, 10 (50 %) responded: 5 with PR and 5 with SD for ≥24 weeks. Seven out of 14 participants (50 %) receiving MCT-CX in the first- or second-line responded, and eleven out of 22 (50 %) responded beyond the second-line. Median PFS was 16 months (Fig. 1), and median OS was 21 months across all participants. (Fig. 2).

### 3.3 Safety

The most reported toxicity was hand-foot syndrome attributed to capecitabine with 39 grade 1–2 events and two grade 3–4 events in 15 of the participants. Gastrointestinal low-grade disturbances included both diarrhea (10 events in 6 participants) and constipation (12 events in 3 participants) were reported. There were two events of cystitis attributed to cyclophosphamide treatment, both were low-grade. (Table 3). These toxicities were managed with dose reductions and no patient discontinued treatment. Dose reductions were also performed due to haematological side effects where white blood cell counts were below 2.5 cells/μL (11 participants). However, no neutropenic infection was observed.

**Table 3:** Toxicity events in a cohort of 40 patients with metastatic breast cancer treated with metronomic chemotherapy with capecitabine and cyclophosphamide.

Grade	Constipation	Cystitis	Diarrhea	Hand-foot syndrome
**1–2**	12	2	10	39
**3–4**	0	0	0	2

There were no serious adverse events. One participant was admitted to the hospital after falling at home and was diagnosed with atrial fibrillation and infection. The patient recovered but discontinued MCT-CX.

### 3.4 Quality of life

**Fig. 3:**
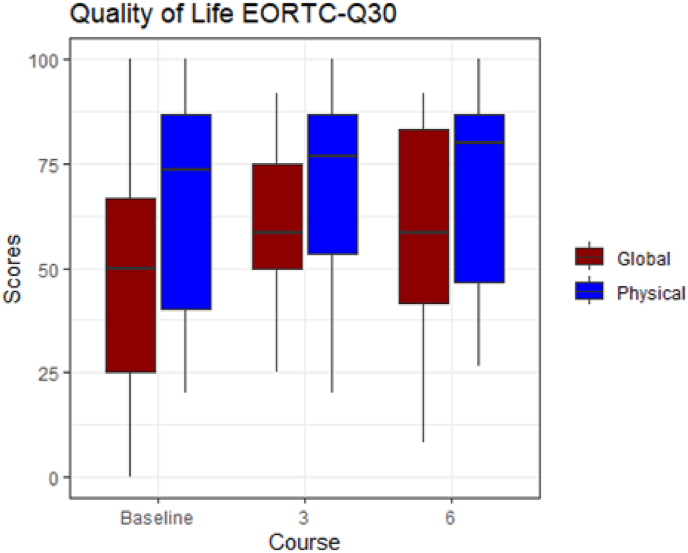
Outcomes of QoL in a cohort of 40 patients with metastatic breast cancer treated with metronomic chemotherapy with capecitabine and cyclophosphamide.

Patient reported QoL measured with the EORTC QOQ C30 questionnaire did not change significantly, either physical or global scores, from baseline to three (p = 0.35, p = 0.10) or six (p = 0.51, p = 0.13) months. At baseline, the mean score for global health status was 50 (interquartile range 25–67), which is at a moderate level. However, the mean physical functioning score was 73 (interquartile range 40–87) which is relatively high, and consistent with participants being in the far advanced stage of the disease. (Fig. 3).

## 4 Discussion

Our findings demonstrate that MCT-CX is an effective and well-tolerated treatment that does not impair QoL in patients with far advanced MBC. This prospective phase II study met its primary endpoint with a CBR of 45 % in pre-treated patients. The low frequency of toxicities, mainly low-grade, and the fact that only one patient discontinued treatment because of toxicity show that MCT-CX is well tolerated. A strength of the study is that we report impact on QoL, it is reassuring to note that there was no impairment during treatment.

Patients have a wish to be asked about their concerns in treatment selection and wish for the oncologist to describe the disease and available treatment options [11]. Research suggests that the patients' perspectives might differ from the oncologists'. Efficacy and side-effects are important for both, but patients reflect wider on how treatments will affect cognition, emotions, and ability to take responsibility for themselves and their family [12]. In a qualitative interview study with patients having experience with MCT-CX, performed within the research group, it was found that study participants appreciated the treatment as a convenient treatment enabling a life as normal as possible despite incurable cancer [13].

The outcomes in the METRO-trial are comparable to earlier studies on MCT in patients with MBC where typically stable disease can be reached but CR and PR are rare [[14], [15], [16], [17], [18]]. For example, the first clinical study with MCT using cyclophosphamide and methotrexate including 64 patients reported an overall clinical benefit rate of 31.7 % [19]. During the last years three studies on different MCT-schedules have shown conflicting results. In a randomized phase II trial, the MCT regiment with weekly non-pegylated doxorubicin combined with oral continuously cyclophosphamide was superior to the same agents given at higher doses every three weeks with ORR of 50 % vs 43 %. In a single arm phase two trial with 29 participants MCT with navelbine and capecitabine showed promising results with an ORR of 31 % and a CBR of 62 % [20]. In contrast, in a randomized phase II trial with 55 participants comparing MCT with cyclophosphamide and Methotrexate with or without bevacizumab ORR was only 26 % and 10 % respectively [21]. As noted above, MCT refers to chemotherapy administered at lower doses, but is not clearly defined and can involve different agents and scheduling.

A commonly used agent MCT regiments is capecitabine. It is recommended as a treatment option in patients with MBC requiring chemotherapy who had previous treatment with anthracyclines and taxanes [2]. However, the optimal dosing of capecitabine has not yet been determined. In a pivotal study from 2011, capecitabine dosed either continuously or intermittently was compared to combination chemotherapy with CMF (cyclophosphamide, methotrexate and 5-FU) in patients with MBC. No significant differences in PFS were seen between the capecitabine arms and the CMF arm, 6 months vs 7 months. Yet, patients in the capecitabine arm had a longer duration of therapy and had a significantly longer OS than those in the CMF arm (22 vs 18 months) with no difference between the two capecitabine dosing schedules [22]. In a recent randomized study, the American Food and Drug Administration (FDA) approved capecitabine dosing of 1250 mg/m2 twice daily, for 14 days, followed by 7 drug free days, was compared to a fixed dose of capecitabine 1500 mg twice daily for 7 days followed by 7 drug free days. The fixed-dose capecitabine had less toxicity and patients in the two treatment arms had similar OS [23]. This indicates that the optimal dosing of capecitabine still needs to be determined. The result of our study suggests that the dosing of capecitabine in the MCT-CX regimen is well tolerated without compromised efficacy.

It is desirable to find ways to better select patients with MBC suitable for treatment with MCT. As expected, clinical variables such as high age, diminished performance status and previous treatment with taxanes has been shown to be associated with worse outcomes of MCT [24]. An easily available biomarker to predict response would be helpful. The molecular mechanisms of MCT were without the scope of this study but it has been suggested that the effectiveness is contributed not only to the direct cytotoxic effects but also indirectly by affecting drug-resistance, activation of immune response and antiangiogenic effects [25,26]. There are different resistance mechanism of fluoropyrimidines (5-FU), the chemotherapeutic agent in capecitabine, depending on dosing schedule [27]. It has been demonstrated that MCT with cyclophosphamide reduced regulatory T-cells and enhanced T and NK-cell functions in blood suggesting a restored immune response in a cohort of end stage cancer patients [28]. Elevated circulating levels of vascular endothelial growth factor (VEGF) as a marker for antiangiogenic effects in patients with MBC treated with MCT has been shown to predict improved disease-free and overall survival [29]. Thymidine phosphorylase (TP) is an enzyme suggested to be a pro-angiogenic factor. Elevated expression of TP in tumour tissue in patients with MBC treated with MCT has been associated with worse outcomes [24]. However, high TP expression has also been proved to be a predictive factor for benefit from fluoropyrimidine containing chemotherapy such as capecitabine. It has been suggested that this is contributed to the crucial role of TP in the conversion of the pro-drug capecitabine to the active antimetabolite 5-fluorouacil in tumour tissue [30]. Since TP has been measured in tissue it is not easily available as a biomarker. If upregulated levels of TP in tumour tissue is reflected in ctDNA, CTCs or extracellular vesicles, widely studied as potential liquid biopsies and can be used as a predictive biomarker for MCT-CX needs to be to be further investigated.

The results of MCT need to be viewed in comparison to more dose intense regiments with taxanes and anthracyclines the most used chemotherapeutic agents in MBC. In an early meta-analysis from 2008 comparing these agents given in first-line median PFS in the whole cohort was 7.1 months and median survival was 19.3 months [31]. Several trials have also reported outcomes on pre-treated patients with MBC. In the EMBRACE-study, where heavily pretreated patients received eribulin or treatment of physician's choice (TPC), the median PFS was 3.7 months with eribulin and 2.2 months with TPC. In the recently published randomized Tempo Breast study the median PFS was 6.6 months with weekly vinorelbine compared with 4.0 months with metronomic vinorelbine [32].

The efficacy of MCT-CX in our study is comparable with these results. However, given that the METRO-trial was non-randomized, there is a potential risk for selection bias wherein most patients were unfit for conventional doses of chemotherapy i.e., MTD. This could make it challenging to compare the results of our study to those of standard dosing chemotherapy. On the other hand, the strict inclusion criteria often seen in clinical trials makes it difficult to generalize the results to the patients commonly seen by oncologists in daily practice. Another limitation is that 38 % of participants had non-measurable disease making it difficult to determine if the disease truly responded.

Further, it is possible that the risk of toxicity has been underestimated by the oncologists since MCT is considered mild and the study was open-label. Another limitation is the small, heterogenous study population. However, the broad inclusion criteria were applied with a pragmatic approach since MCT-CX is commonly used in frail, pre-treated patients who are often not included in RCTs.

In summary, this study describes a population of patients with far advanced MBC commonly seen by oncologists in daily practice. Our findings suggest that MCT-CX is an efficient treatment option that could minimize QoL disturbances for patients during the remainder of life. However, further studies comparing MCT-CX with chemotherapy given at the MTD as well as studies comparing MCT-CX to best supportive care are needed.

## Funding

The study was financed by grants from the Swedish state under the agreement between the Swedish government and the county councils; the ALF-agreement, Anna-Lisa och Bror Bjornssons foundation, Brostcancerforbundet and the Sahlgrenska University hospital foundation.

## CRediT authorship contribution statement

**Karolina Larsson:** Writing – review & editing, Writing – original draft, Visualization, Resources, Project administration, Methodology, Investigation, Funding acquisition, Formal analysis, Data curation. **Jamila Adra:** Writing – review & editing, Investigation. **Leif Klint:** Writing – review & editing, Investigation. **Barbro Linderholm:** Writing – review & editing, Supervision, Methodology, Investigation, Funding acquisition, Conceptualization.

## Declaration of competing interest

Dr Linderholm reported receiving unrelated advisory funding from AstraZeneca, Daiichi-Sankyo, Gilead, Pfizer, Eli Lilly, Novartis. Dr Larsson reported unrelated funding from Pfizer, Dr Klint reported unrelated advisory funding, Dr Adra did not report any COIs.
