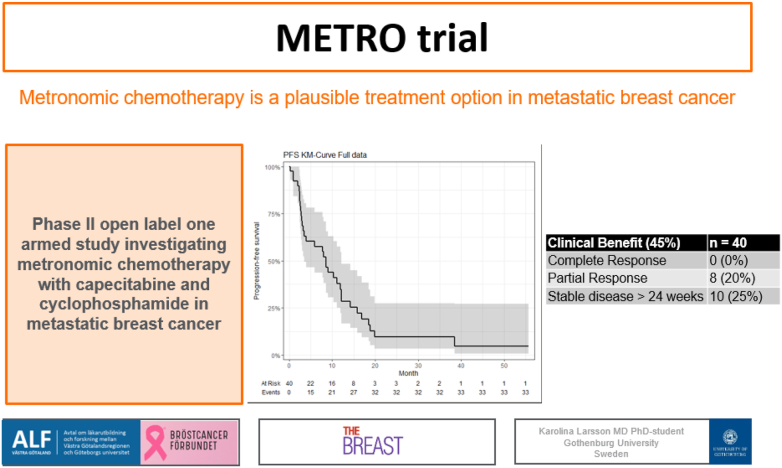# Corrigendum to “Metronomic chemotherapy using capecitabine and cyclophosphamide in metastatic breast cancer – efficacy, tolerability and quality of life results from the phase II METRO trial” [The Breast 78 (2004) 103795]

**DOI:** 10.1016/j.breast.2025.104420

**Published:** 2025-03-13

**Authors:** Karolina Larsson F, Jamila Adra, Leif Klint, Barbro Linderholm

**Affiliations:** a Department of Oncology, Sahlgrenska University Hospital, Sweden; b Institute of Clinical Sciences, Sahlgrenska Academy, University of Gothenburg, Sweden

The authors wish to correct an error in the reported median progression-free survival (PFS) in our study. Due to a misunderstanding, the date of death was mistakenly used instead of the date of progression. As a result, the originally reported median PFS of **16 months** is incorrect. The correct median PFS is **8.5 months** as indicated in the updated Fig. 1 and the Graphical Abstract.

As PFS was only a secondary outcome, we believe this correction does not impact the main conclusions of the study.

The authors would also like to acknowledge Ahmet Akdeve at Statistikkonsulterna Väst AB for their prompt assistance in correcting the statistical error.

We sincerely apologize for any confusion or inconvenience this may have caused.

**Fig. 1:**
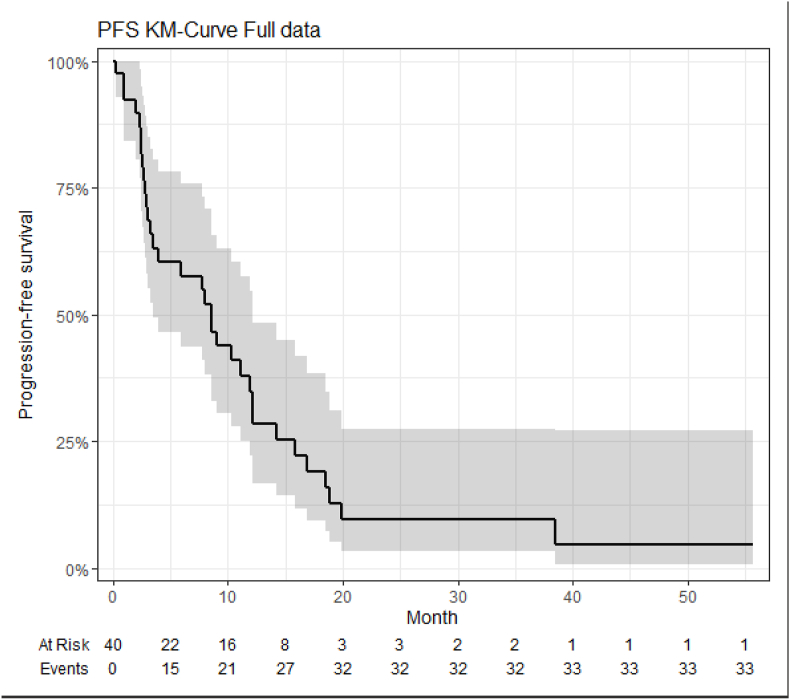
PFS in a cohort of 40 patients with metastatic breast cancer treated with metronomic chemotherapy with capecitabine and cyclophosphamide.

## Graphical abstract

**Figure:**