# Supplementary material for: Metronomic chemotherapy using capecitabine and cyclophosphamide in metastatic breast cancer – efficacy, tolerability and quality of life results from the phase II METRO trial
Source: Breast. 2024 Sep 1;78:103795. doi: 10.1016/j.breast.2024.103795 (PMC11404084; doi:10.1016/j.breast.2024.103795)
Supplement: Multimedia component 1 [file mmc1.docx]

Inclusion and exclusion criteria METRO-trial

**Inclusion criteria**

1. Written informed consent approved by the Independent Ethical Board.

2. Female or male patients ≥18 years with breast cancer confirmed by histology.

3. Recurrence (local or distant) not possible to cure.

4. No or any line of previous therapies for recurrent disease. or 1^st^ line chemotherapy for recurrent disease accepted. If other types of chemotherapy are given previously, an interval of ≥ 4 weeks between last administration and inclusion in the present study is recommended.

5. Measurable or evaluable disease.

6. Life expectancy of ≥3 months

7. Adequate bone marrow, renal, hepatic and cardiac functions as judged by the responsible doctor and no other uncontrolled medical or psychiatric disorders.

8. ECOG performance status 0-2.

9. Patients in child-bearing age must have adequate contraception.

10. Patient willing to participate in blood sampling according to **APPENDIX** **II**.

**Exclusion criteria**

1. Clinically significant (i.e. active) cardiovascular disease.

2. Non-healing wound, active peptic ulcer or bone fracture.

3. Evidence of any other disease, neurological or metabolic dysfunction, physical examination finding or laboratory finding giving reasonable suspicion of a disease or condition that contraindicates the use of an investigational drug or puts the patient at high risk for treatment-related complications
